# Moral Philosophy; or the Duties of Man Considered in His Individual, Domestic, and Social Relations

**Published:** 1841-10-01

**Authors:** 


I.—Moral Philosophy; or the Duties of Man considered in his Individual, Domestic, and Social Relations. By *George Combe.* 8vo. Edinburgh and London, 1840, p. xvi. 440.

II.—Notes on the United States of North America, during a Phrenological Visit, in 1838-9-40. By *George Combe.* Three volumes, 8vo. Edinburgh and London, 1841, p. xxiv., 1260. With a Map and Illustrative Diagrams.

[Continued from No. lxix., p. 80.]

II. The Notes on the Americans.

These Notes are exceedingly multifarious; and, in that they are distinguished by much faithfulness of observation as well as an exemplary impartiality of judgment, they cannot fail of proving unusually attractive to many persons, although induced to examine the author's views with very different dispositions and purposes. Here, in Mr. Combe's entertaining and instructive pages, the agriculturist and commercialist, the legislator and moralist, the philosopher and artisan, the civilian and religionist, the physiologist and physician, the phrenologist and philanthropist, will all find abundance and diversity of congenial topics whereon to exercise their practical and contemplative powers. We prefer the subsequent gleanings, as not unsuited to the pages of this journal: let us begin, then, with "Notes" on Insanity, Lunatic Asylums, and Medical Jurisprudence.

Having read, in the American Jurist, No. xxxviii, a very able review of Dr. Ray's Treatise on the Medical Jurisprudence of Insanity,* Mr. Combe there found it recommended to public attention, on account of the talent which it displays, also because it treats of insanity, on phrenological principles, and because it embodies the views of the most recent French, British, and American authorities on this exceedingly important subject. He afterwards consulted the work itself; and, as he has rightly judged, it contains much excellent matter. In an able essay prefixed to the volume, he says, the contradictions, errors, and inhumanity of the doctrines on insanity delivered by the lawyers of England, up to a very recent date, are clearly stated and completely exposed. Nor does Scotland with her groupes of sages in the moral and metaphysical sciences, escape Dr. Ray's enlightened and searching scrutiny. He observes at p. 50, the doctrine of *moral* insanity has been as yet unfavourably received by judicial authorities, not certainly for want of sufficient facts to support it, but probably from that common tendency of the mind to resist innovations upon old and generally received views. Since, a quarter of a century ago, one of the highest law-officers in Britain pronounced the manifestation of "*systematic correctness*" of an action a proof of sanity sufficient to render all other evidence unnecessary, it is not surprising that the idea of *moral* insanity has been considered by the legal

* An Analytical Account of Dr. Ray's Treatise was presented to our readers, in the No. of this Journal for April, 1839, p. 431-443.

profession as a fancy sprung from the teeming brains of medical theorists. Thus, in the fulness of this spirit, Mr. Chitty declares that, "unless a jury should be satisfied that the *mental faculties* have been *perverted*, or at least the faculties of *reason and judgment*, it is believed that the party subject to such a *moral* insanity, as it is termed, would not be protected from criminal punishment;" * and, Dr. R. continues, in the trial of Howison for the murder of the widow Geddes in Scotland, *moral* insanity, which was pleaded in his defence, was declared by the court to be a groundless theory. Such opinions, from quarters where a modest teachableness would have been more becoming than an arrogant contempt for the results of other men's inquiries and experience, involuntarily suggest to the mind a comparison of their authors with the saintly persecutors of Galileo, when they resolved, by solemn statutes, that Nature always had operated, and always should operate, in accordance with their views of propriety and truth. Many, very many facts might be adduced to corroborate these incontrovertible observations of Dr. Ray's, and to evince the excessive dulness and dogmatism generally displayed in the jurisprudential proceedings regarding insanity, throughout this country. In fact, there is no consistent or intelligible definition of insanity in the forensic rules and authorities of Britain; and, therefore, in consequence of this legal anarchy, the judgment of cases is too often a judgment of despotism, a mere opinion emanating from a mind bewildered by prejudice and enchained with the fetters of ignorance and bigotry.

Adverting to the indecent haste with which the trial, sentence, and execution of Bellingham, for shooting Mr. Percival, were hurried over, Dr. Ray remarks, that few will now read the report of this trial without being forced to the conclusion, that Bellingham—was really mad; or, at the least, as Mr. C. adds, that the case should have been deliberately investigated. Again, with reference to Howison's case, the doctor adds, that application was made to the secretary of state, by the law-agent of the accused, for time to obtain further evidence of his insanity, but without success, although several post-judicial facts were added, and left no doubt that the unhappy convict was not a fit subject for mortal punishment. From actual knowledge, Mr. C. confirms these statements, and produces the additional information—that, in the night preceding Howison's execution, he made a confession of a number of murders which he avowed himself to have committed, and of which he specified the times, places, and circumstances, evidently believing them to be real; but which, on inquiry, one and all of them turned out to be mere phantoms of his own diseased brain. In this wretched man, the innate propensity to destructiveness appears to have been liable to accessions of inordinate excitement giving rise to destructive monomania; and, while labouring under one of these paroxysms, which

---

* Chitty, Medical Jurisprudence, p. 352. As Mr. C. prefers the superannuated terminology of the metaphysicians who convert reason and judgment into mental faculties, he might have recollected that these oracular sages also represent the "*Moral Sense*," as being a mental faculty; and, consequently, he could scarcely have failed to see, if he could not acknowledge, that perversion of this prominent faculty should be held for a *moral* insanity.—*Rev.*

misled his own judgment and memory, and prompted him to clothe its suggestions with the attributes of reality, he was led forth to the gallows and executed. We agree entirely with Mr. Combe in the opinion that, from the evidence adduced at the trial and subsequently obtained, this man Howison undoubtedly committed the homicide during a monomaniacal state of mind, without provocation, and without any motive discernible by a sane understanding.—When will our prominent patriots cease from expending their energies in toiling for the attainment of superficial reforms! Let them rather cultivate the principles and their applications requisite for maturing education and legislation; and, on these being perfected by a just and consummate reformation, that of all other institutions will necessarily follow: and, from that day forth, we shall no longer be harassed with the horrors of contemplating the destruction of maniacs, executed as criminals.

At p. 196 of Mr. Combe's first volume, we meet with "Notes" from a valuable official report on insanity and criminal jurisprudence. Chief-Justice Parker delivered a charge to the grand jury for Merrimack county, in 1838, on the subject of insanity; and, in this charge, which was published by request, the phrenological views of this malady were embodied. From this report, we learn that, by returns to the legislature of New Hampshire, in 1833, there were 193 cases of insanity in eighty-three towns; while, from one hundred and twenty-seven towns, no report was received. Hence, at a similar ratio for all the towns in the state, the number would be about 500 cases: of those *returned*, 98 were paupers and 95 not so: neither the local nor general population is recorded. From the returns, it is shown that about a half was, or had been, in confinement: some were in cages and cells, some in irons and chains, and some in jails.

Further, we observe in the report of a Committee, in 1836, that one hundred and sixty-one towns had furnished returns; that, in one hundred and forty-one of these towns, there were 312 cases of insanity; and that, in the remaining twenty, no case was specified. According to the same document, the period in which insanity existed, was from two weeks to sixty years, and gave an average of 13½ years' duration. Taking the ratio of the population *(which is not stated)* of the towns that made returns, as compared with the population of the state, the result would show that they included nearly 450 insane persons; but, says the report, there are obvious reasons why this should be below the actual number. By inquiries recently instituted, we discover the fact—that, in the county of Chester, *U.S.*, for every thousand of the inhabitants about two are insane. Mr. Parker next proceeds to distinguish the various circumstances in which the question of insanity may come before courts of law and juries: such as, claims for the support of lunatics—applications for the appointment of guardians to lunatics—controversies about wills executed by persons alleged to have been insane—disputes about contracts said to have been undertaken by the insane—and discussions concerning the guilt or innocence of persons represented as labouring under insanity at the time of committing an offence. He then gives a description of the disease, in which he recognizes not only *intellectual* insanity, but also morbid affections of the *feelings*. He expressly says that "the *propensities* and *senti-*

*ments* may become deranged ;" and, among other forms of their diseases, he includes an "irresistible propensity to steal," an "inordinate propensity to lying," a "morbid propensity to incendiarism," and a "morbid propensity to kill." He illustrates his divisions of insanity by quotations from the reports of Dr. Woodward, superintendent of the Worcester (U. S.) Lunatic Hospital, who is an avowed phrenologist, and from Dr. Ray's work on medical jurisprudence. It is gratifying, as Mr. Combe remarks, to see the principles of phrenological science thus brought lucidly and practically to bear on the interests of a state, under the direction of one of its highest authorities.

The reader would observe our allusion to the Worcester Lunatic Hospital and its superintendent: we will here introduce some account of them. Mr. Combe's picture of the one, and his portrait of the other, is exceedingly graphic. This monument of charity erected by the State of Massachussetts, at an expense of eighty-five thousand dollars, is situated on a beautiful eminence eastward of the town. The buildings of the west front, erected in 1831, consist of a centre, 76 feet long, 40 wide, and four stories high, projecting 22 feet forward of the wings. These extend to the north and south 90 feet each on the front, and 100 feet in the rear, and are 36 feet wide, and three stories high. This arrangement was adopted to secure free communication with the central structure, occupied by the superintendent, steward, attendants, and domestics, and to permit the ventilation and lighting of the long halls, or corridors, extending through the wings. The apartments for the insane, 8 feet by 10, have each a window, with the upper sash of cast-iron and lower of wood, both glazed; on the exterior of the wooden sash is a false sash of iron, corresponding to it in its appearance and dimensions, but firmly set into the frame of the window, giving the reality of a grating without its gloomy aspect. In 1835, a building 134 feet in length, and 34 feet in width, was attached to the southern extremity of the hospital, of equal height, and extending eastward at right angles with the front; in 1836, another edifice of the same magnitude was erected at the north end. Three sides of a great square are now inclosed by these extensive structures of brick.

This hospital was opened in January, 1833; from the first it has been admirably managed and the system of moral and medical treatment attended with great success. The structure itself combines the improvements which have recently been introduced into hospitals for the insane. It commands a cheerful and even beautiful prospect, from every window occupied by the patients. The ventilation and heating of the rooms are accomplished by warm air introduced into the galleries, and from them into each cell, by means of an oblong opening above the door. The pipes conveying hot air open near the ceilings of the galleries, and some advantages attend this arrangement. If the pipe opens at the floor, the stream of warm air does not diffuse itself over the apartment, as is generally supposed, but ascends in a direct column to the ceiling and is only there broken and dispersed. It descends only after it has filled the upper spaces of the galleries. By introducing it at once at the top, these spaces are filled before much of the heat has been lost, and a warmer air descends, and enters the several rooms by the apertures above the doors. In the wall of each room is an opening about five inches square, into an airchimney, calculated to maintain a constant circulation. These air-chimneys open into a vast garret, directly under the roof of the building, which contains numerous windows for letting off the noxious air into the atmosphere. This arrangement is attended by one considerable advantage. When the air-chimneys open directly into the external atmosphere, their action is violently affected by the state of the wind; and, in cold weather, they bring down cold instead of carrying up heated and exhausted air. When they open into the garret, they are altogether protected from the wind; and by opening fewer or a greater number of the garret windows in Winter, effectual security is obtained against the descent of cold air. These chimneys are effectual; and, in the morning, the rooms are pure to the senses, while the garret furnishes abundant evidence that it has received the effluvia of the night. At the end of the galleries, there are two large corner apartments, two sides of which are composed entirely of cast-iron sashes, one for the women with the interstices glazed; the other for the men without glass. The object of these is to afford air and exercise to the patients in severe weather when they cannot go abroad, or when their state of health renders complete exposure inexpedient. There is abundance of ground attached to the hospital, in which the patients labour, and there is a chapel in which divine worship is performed every Sunday. A neat carriage, drawn by two horses, belongs to the establishment, and is constantly employed in carrying the patients little excursions into the country to amuse them. The purity and order of the apartments are complete.

We wish to make our readers acquainted with Dr. Woodward, the superintendent of this Hospital, by presenting them with Mr. Combe's portraiture of this justly distinguished physician. Physically and mentally, says our experienced limner, Dr. W. is admirably adapted for his situation. He is in the prime of life, and has large limbs, a large abdomen, large lungs, and a large head. His temperature is sanguine nervous-bilious, with a little of the lymphatic. The organs of the propensities are well developed, but those of the moral sentiments and intellect decidedly predominate. This combination produces a powerful and commanding person, characterized at once by vivacity, energy and softness; and a mind in which intellectual power is chastened by the most kind and cheerful moral dispositions. Mr. C. regards these qualities as of great importance in the superintendent of a lunatic asylum. If that well-spring of spontaneous vivacity which accompanies large lungs and a large brain be wanting, the individual will be more apt to sink under the depressing influence which the diseased minds of his patients will exert over his own, than to excite their faculties to more healthy and agreeable action. If he be deficient in the moral organs of the brain, he will want sympathy, softness of expression, and justness of feeling; while if he be deficient in the reflecting intellectual organs, he will want sagacity to trace effects to their causes, and to discriminate character; or, if the deficiency be in the observing organs, he will lack the power of attention to incidents and details.

Dr. Woodward has published a valuable pamphlet, strongly urging the advantage of instituting "asylums for inebriates." His reasoning may be briefly stated thus: "1. Intemperance is a physical disease. 2. It is curable in the great majority of cases, if not always. 3. The greatest existing difficulty in effecting this end, commonly arises from the extent of the temptation to which the patient is uniformly exposed. 4. The best remedy for this state of things is to confine the individual, with a view to the avoidance of this temptation, and to the adoption of whatever other measures are necessary for this cure—till he is cured—under charge of an institution expressly adapted to the purpose." The subject has attracted considerable attention in the United States; and, as Dr. Woodward's views are unquestionably sound, both physiologically and morally, it is to be hoped that his suggestions may be extensively carried into effect.

Our traveller had the good-fortune to accompany Mr. Salisbury, a state inspector, in his official visitation of the hospital at Worcester: and here, while transcribing his "Notes," we would direct attention to his remarks on the house and its internal economy. Being invited to accompany the inspector, Mr. C. entered every cell and apartment; he saw every patient in the institution; and, in his deliberately recorded opinion, nothing could exceed the excellent condition in which it appeared. Only five furious and filthy patients were found among the whole, and they are lodged in a separate building, so distant that their noise cannot annoy the general inmates of the hospital. Each of these persons was in a distinct cell, the walls of which are of brick, and the floors of mica-slate pavement, heated by fire applied below. The light is admitted from the passage. In one of the cells was a musician, who tears everything to pieces, and is excessively dirty. He was seated on the warm stone-floor, clothed in a very strong and thick cotton vestment, which descended to his ankles. His organs of time and tune remained sound amidst the wreck of nearly all his other faculties. While thus seated, he played several tunes on the flute, with correctness and expression. His head is well formed, with the exception of a predominating destructiveness. His temperament is nervous-sanguine, and the organs of imitation and ideality, as well as those of time and tune, are largely developed. Dr. W. gave the patients of the hospital a ball on Christmas Eve. They themselves decorated very tastefully one of the corridors, with boughs of evergreens, and converted it into a handsome ball-room. They looked forward to the entertainment with great interest for many days before Christmas, and it still afforded them a pleasing theme of conversation. It proved very successful, and even this musician performed a part in it. Dr. W. is an enlightened phrenologist, and he assured Mr. C. that his conviction increases, the more he observes, that the cases are extremely rare in which the whole of the mental organs are involved in disease; and that this conviction led him to try the experiment whether this individual could not be enabled to command himself at the ball. He explained to him the preparations that had been made; asked him if he would like to attend. This wakened up a thousand impressions, received in his best days of health and usefulness, and he professed his desire to assist and to play in his professional capacity. Dr. W. adverted to his dress, and said that he must appear in the costume of a gentleman, and must conduct himself with decorum, as the only conditions on which he could be admitted. He engaged to comply with both stipulations. When all things were prepared on the evening of the ball, the keepers entered his cell, dressed him in a decent suit of clothes, and led him to his seat among the musicians, and instantly the band struck up, and the dancing commenced. He played in perfect tune and time. One of the keepers was stationed behind him all the evening to prevent accidents, in case of his losing command of himself; but there was no need for his interfering. For three hours, he continued to play and conduct himself with perfect propriety, At the end of two hours, he complained of fatigue, and said that he believed that formerly he used, about this time, to receive a glass of wine. A glass of wine was given to him, he drank it, and played on, till the close of the entertainment. He was then reconducted to his cell, and had hardly entered it, when he recommenced tearing his clothes.

Dr. Conolly, in his instructive Report on the Hanwell Lunatic Asylum for MDCCCXL., observes—that "the principles of changing all the circumstances surrounding a lunatic, is evidently one capable of application in certain cases, and in certain periods of the malady, with singularly felicitous effects." We have always regarded this as a fundamental principle in the management of insanity, and we could instance, within the range of our own observation, the fatal results which have been occasioned by ignorance or wilful defiance of this most essential principle. In the deeply affecting and deceptive forms of transitory monomania which is so prone to arise in delicate and excitable females during pregnancy or convalescence after child-birth, no resource can be more preposterous and pernicious than that of secluding the patient in the bosom of her family, surrounded by rueful relatives, and attended by woe-begone familiars. Such a resource is known to have been adopted; and, from experience, it is also known to be most effectual in depriving the patient of every chance of recovery. In Dr. Woodward's destructive musician, although a confirmed lunatic, we have a remarkable example showing how powerfully a total change of circumstances may affect an insane person even in the most hopeless condition. In this case, the effect was temporary, but it was great while it lasted.

Dr. Woodward informed his visitants that he allows about one-fourth of the inmates of the asylum to go into the village on specific errands unattended, and only one man has escaped; and he did so after being enticed by some acquaintance to drink. Social parties, with music and dancing, are given from time to time, which, with religious worship on Sundays, have an excellent effect on the minds of the patients. The music is supplied entirely by the patients themselves. Mr. C. saw in the hospital a woman, who, in a fit of religious and destructive mania, had attempted to cut off the heads of two of her children. Philoprogenitiveness was deficient, and destructiveness enormously large. A man who is insane in regard to wealth, imagining himself to possess incalculable riches, has the organs of acquisitiveness standing forth in such ample size and well-defined forms, that they attract the eye in looking at him in passing. Ideality is also large, and in his imagination he applies his wealth to gorgeous purposes. There were other striking examples of the concomitance between the peculiar features of monomania and the size of particular organs in the brain; and Dr. W. expressed his surprise how any man, living in charge of an hospital for the insane, and capable of mental analysis and physical observation, reasonably acquainted with phrenology, could avoid conviction of its truth. He also mentioned that he receives many shoemakers as patients. This class is numerous in New England: but he believes that insanity is produced beyond an average extent among them by their breathing vitiated air in their hot, small workshops, without ventilation, and by their unfavourable position when working. Many sailors are admitted into the institution as patients, and their insanity is held to be produced by intemperance and severe hardships at sea. The cures in cases of less duration than twelve months, amount to 85 per cent. on an average of six years.

We are furnished by Mr. Combe with a copious account of the "Eastern Penitentiary" in Pennsylvania, and of the effects of solitary confinement on the health and morals of criminals. This Penitentiary is a state-prison, situated on high ground north-west of Philadelphia, and built in the gothic style. It covers about ten acres of ground, and the ranges of cells radiate from a central tower to the high walls which surround the whole. It owes its origin to "the Philadelphia Society for alleviating the miseries of public prisons," who, from 1801 to 1821, addressed several petitions and memorials to the Legislature, praying for the more effectual employment and separation of prisoners, and for proving the efficacy of solitude on the morals of those unhappy objects. When a prisoner arrives at the Penitentiary, he is examined by the warden, and is then taken to the preparing room. Here he is divested of his usual garments, his hair is closely trimmed, and he undergoes the process of ablution. He is then clothed in the uniform of the prison, a hood or cap is drawn over his face, and he is conducted to his cell. The bandage is removed from his eyes, and he is interrogated as to his former life, which, as a matter of course, is seldom accurately related. The consequences of his crime, the object in view in his punishment, and the rules of the prison, are next explained to him. He is then locked up in solitude without employment. After enduring this state of existence for some days, and feeling its discomfort, he supplicates for the means of employment; which are granted to him, not as a punishment, but as a favour. He is also furnished with a Bible, some religious tracts, and occasionally other works calculated to imbue his mind with moral and religious impressions. In every cell there is a pipe supplying pure water, a kind of water-closet, a bed, a chair, and the implements of the convict's labour. The apartment is heated in Winter by pipes filled with hot water, and there is an aperture for ventilation, which is at the command of the convict. Every convict is obliged to keep his cell perfectly clean, and great attention is paid to the cleanliness of his clothing and person. The men receive a towel, a razor, and shaving apparatus. The clothing is comfortable, and adapted to the season. Their food consists, for breakfast, of one pint of coffee or cocoa, made from the cocoa-nut, or mush. Dinner, three-quarters of a pound of boiled beef without bone, or half a pound of pork, one pint of soup, and an ample supply of potatoes. Occasionally boiled rice instead of potatoes. Supper, mush (made of the flour of Indian-corn boiled) *ad libitum*, one half-gallon of molasses per month, salt whenever asked for, and vinegar as a favour occasionally. Turnips and cabbage in the form of crout are sometimes distributed. The daily allowance of bread is one pound, made of wheat or rye.

For the sake of comparison, Mr. Combe here introduces, in a foot-note, the allowance of food in the Glasgow Bridewell, which he commends as one of the best-managed prisons in Scotland. This bill of fare is copied from the fifth report of the inspectors of prisons, and we give it as follows: Breakfast, eight ounces of oatmeal made into porridge, with a pint of butter-milk. Dinner, two pints of broth (soup), containing four ounces of barley, and one ounce of bone, with vegetables; also, eight ounces of bread. Supper, five ounces of oatmeal, made into porridge, with half a pint of butter-milk. Cost of the whole, 3¼*d*, including cooking. Our readers connected with the poor-law-unions and union workhouses will be able to appreciate the difference between this "allowance," and that usually doled out to paupers unaccused of criminality.

Returning to Mr. C.'s account of the Penitentiary, we find that each convict, on his reception, receives a number in the books, which is marked over his cell-door, and on his clothes. This is his prison-name, and his proper name is kept concealed. This rule prevents one convict from learning the name of another. The convicts on the ground-floor are allowed to walk one hour a day, in a very small yard which is attached to each cell, and the hours are arranged so that no two contiguous yards are occupied at the same time. The cells on the upper floor do not admit of this exercise being enjoyed, but the inmates of them are equally healthy with those who inhabit the cells below. Divine service is performed on Sundays, by pastors who serve gratuitously. The chaplain takes his station at one end of a corridor, the prisoners approach their doors, open a small wicket in them, and listen. A curtain is let down in the centre of the corridor, to prevent the convicts from seeing each other across it. Religious books are supplied, but there is no library of miscellaneous publications. The punishments inflicted for breach of discipline are deprivation of exercise, diminution of the quantity of food, and confinement in a dark cell. No flogging is allowed, and very little punishment of any kind is required. A board of inspectors appointed by the Legislature, exercises a general superintendence over the prison, and reports annually to the Legislature. Their reports are accompanied by reports also from the warden and physician; the whole forming authentic and interesting records of the numbers and condition of the convicts, and of the effects of the Penitentiary system for each successive year.

From a careful examination of the reports on this Penitentiary for 1832, 1833, 1834, 1835, 1836, 1837, and 1838, Mr. Combe found the inspectors earnestly representing to the Legislature, the want of "the services of an experienced, intelligent, and pious man, who shall be the instructor of the convicts, and visit them frequently in their cells, inculcating day by day the principles of temperance and religion." Whatever help they receive towards mental improvement are derived from the gratuitous services of clergymen. In his reports, the warden specifies deficiency of education as one common cause of crime, and he remarks that the convicts in general do not possess the instruction given even in the free-schools of the state. He also repeats again and again, as the result of all his experience —that, "to communicate any material benefit to those who are brought to the Penitentiary, their sentences should extend *to two years or more.*" This extraordinary neglect of moral and religious instruction inspires our philanthropist with a train of extremely just and justly severe reflections.

No single circumstance, he observes, in the history of Pennsylvania, indicates the low state of general information among her people more strongly than the extraordinary fact here brought to light, that, after erecting this Penitentiary at a vast expense, and providing it with all the physical requisites for accomplishing the objects for which it was instituted, the Legislature continues insensible to every entreaty of its legal guardians, urged in the most forcible language for six successive years, to be furnished with adequate means of moral and religious instruction for the prisoners. An enlightened people would as soon have built a palace without a roof, as have instituted a Penitentiary (a house for moral reformation), without a moral and religious instructor! One such teacher is not sufficient. If the intention of improving the minds of the convicts be seriously entertained, labour must be bestowed in the cultivation of their moral and intellectual faculties proportionate to their ignorance and wickedness. If well constituted minds require extensive moral and religious training and instruction to preserve them in the paths of virtue, ill constituted minds need much more. Although we form the highest estimate of the quantity and quality of this instruction furnished to the convicts, by the excellent persons who labour in the Penitentiary gratuitously, or who are rewarded by benevolent societies, the very fact that the legal inspectors proclaim it to be insufficient, leaves the Legislature of Pennsylvania without excuse in denying a further supply. Whenever the philosophy of mind is generally understood, this penury of instruction will appear nearly incomprehensible. The philanthropists of Europe expect the American commonwealths to prove, by a liberal expenditure of public money for *moral* objects, the superiority (of which they so loudly boast) of a government emanating from, and responsible to, the people, over those which depend on the will of an individual or of a high-born aristocracy. A democracy which refuses moral and religious instruction to its convicts, apparently from no consideration except that of saving the expense, is a greater foe to freedom than the most ruthless despot of Europe: such a democracy saps the faith of good men in human virtue; while the tyrant only stifles its outward manifestations, leaving the faith itself to burn the brighter the more he labours to extinguish it.

In his seventh report, the warden observes that "a minute inspection of the character of the unhappy inmates of prisons has developed another interesting fact—that many more of them than was supposed are really irresponsible beings;" and, to the same effect, the inspectors remark, that "there are no doubt *some criminals* who *are incorrigible.*" On such, the effects of the Penitentiary system do not generate vindictive feelings, but they leave the establishment with sentiments of regard rather than resentment towards those who have attempted to alter their vicious habits. Phrenologists have long proclaimed, that the great cause of the incorrigibility of criminals is the excessive predominance of the organs of the animal propensities, over those of the moral and intellectual faculties, and that this class of persons is really composed of moral patients, who should be restrained, but not otherwise punished, during life. As Nature is constant in her operations, this truth will in time force itself on the conviction of society; and after injustice and severity shall have been perpetrated for ages, by the free and the fortunate towards the ill-constituted and unhappy, a better system of treatment will probably be adopted.

The health of the prisoners is indicated, by Mr. C. in a tabular view which gives 3.4 per cent. as the average of deaths. In some instances, according to the Reports, the deaths arise from incurable disease affecting the prisoners at their entry, and that the average is greatly augmented by the sickly inefficient condition of the coloured prisoners, who "by self-abuse, become debilitated in mind and body, and diseased, and make up 3-5ths of the whole mortality."

A few convicts, labouring under insanity at the time of their condemnation, have been sent to the Penitentiary, and the inspectors complain that in one or two instances they have been convicted in the full knowledge of their insanity, with a view to get quit of them as troublesome to the country. This fact is not a little surprising: the next has an important bearing on prison-discipline. In a report read to the Senate on February 14, 1837, a committee of the legislature stated, that "no instance of insanity has, as yet, occurred in the Eastern Penitentiary, which has not been traced to causes wholly independent of, and either anterior or posterior to the confinement. Whatever might be the disturbing and stultifying effects of strict seclusion, without labour, without books, without moral instruction, and without daily intercourse with the keepers, certain it is, that with all these circumstances to relieve the distressing ennui, and the supposed maniacal effects of absolute isolation, the inmates of our prisons are in no danger of mental aberration, or alienation of mind, from the cause supposed:" it is "shewn conclusively that the unbroken solitude of the Pennsylvania discipline does not injuriously affect the health of the convicts." Mr. C. visited a number of the male convicts who had been confined for periods ranging from seventeen months to eight years, and their appearance did not indicate either bad health or mental depression. He was introduced also into the cells of several female convicts, some of whom had ornamented the walls with pictures and needlework, giving to the apartments an appearance of tidiness and comfort that bespoke a healthy condition of mind in the inmates. To him, the food appeared to be too rich and abundant for solitude, and several of the men had applied to be placed on a tea diet, consisting of tea and bread, which is allowed them when asked for. Secret vice abounds among the men, particularly the coloured convicts, who have few mental resources; but one of the white male prisoners had celebrated its pleasures and pains in an ode written with a pencil on the white-washed wall of his cell.

We will now introduce briefly Mr. C.'s own opinion concerning the effects of systematic seclusion in this Penitentiary. He observes that the system of entire solitude, even when combined with labour, and the use of books, and an occasional visit from a religious instructor, leaves the moral faculties still in a passive state, and without the means of vigorous active exertion. According to his view of the laws of physiology, this discipline reduces the tone of the *whole* nervous system to the level which is in harmony with solitude. The passions are weakened and subdued, but so are all the moral and intellectual powers. The susceptibility of the nervous system is increased, because organs become susceptible of impressions, in proportion to their feebleness. A weak eye is pained by a degree of light which is agreeable to a sound one. Hence, it may be quite true, that religious admonitions will be more deeply felt by prisoners living in solitude, than by those enjoying society; just as such instruction, when addressed to a patient recovering from a severe and debilitating illness, makes a more vivid impression than when delivered to the same individual in health; but the appearances of reformation founded on such impressions are deceitful. When the sentence is expired, the convict will return to society, with all his mental powers, animal, moral, and intellectual, increased in *susceptibility*, but *lowered in strength*. The excitements that will then assail him, will have their influence doubled, by operating on an enfeebled system. If he meet old associates and return to drinking and profanity, the animal propensities will be fearfully excited by the force of these stimulants, while his enfeebled moral and intellectual powers will scarcely be capable of offering any resistance. If he be placed amidst virtuous men, his higher faculties will feel acutely, but be still feeble in executing their own resolves. Convicts, after long confinement in solitude, shudder to encounter the turmoil of the world; they become excited as the day of liberation approaches, and feel bewildered when set at liberty. In short, this system is not founded on, nor in harmony with, a sound knowledge of the physiology of the brain, although it appeared to Mr. C. to be well administered.

Solitary confinement, with labour, is inforced at the New Jersey state prison, and the preceding views are strengthened by the report presented to the Board of Inspectors in 1839, by Dr. J. B. Coleman, the physician to this institution. He states that, among the prisoners, there are many who exhibit a child-like simplicity, which shews them to be less acute than when they entered. In all who have been more than a year in prison, some of these effects have been observed. Continue the confinement for a longer time, and give them no other exercise of the mental faculties than this kind of imprisonment affords, and the most accomplished rogue will lose his capacity for depredating with success upon the community. The same influence that injures the other organs will soften the brain. Withhold its proper exercise, and as surely as the bandaged limb loses its power, will the prisoner's faculties be weakened by solitary confinement. Dr. C. concludes his account of the effect of this treatment, in these terms: "While it subdues the evil passions, almost paralyzing them for want of exercise, it leaves the individual, if still a rogue, one who may be easily detected;" in other words, in reducing the energy of the organs of the propensities, it lowers also that of the organs of the moral and intellectual faculties, or causes the convict to approach more or less towards general idiocy. Dr. C. does not inform us whether the brain will not recover its vigour after liberation, and thus leave the offender as great a rogue after the close, as he was at the beginning of his imprisonment.

We have dwelt at greater length on this Penitentiary and its discipline, then was our original intention; but, as we see great importance in the records of principles and system in the experience of intelligent medical officers, so we feel prompted to assist still farther in enabling medical officers at home to appreciate the results of "solitary confinement" under the direction of their transatlantic brethren. With this impression for our inducement, we would beg the reader to accompany us a little longer in attending to Mr. Combe's concluding observations relating to this establishment. At Auburn, there is a system of social labour which, in his opinion, is better than that of Pennsylvania, in so far as it allows of a little more stimulus to the social faculties, and does not weaken the nervous system to so great an extent; but it has no superiority in regard to providing efficient means for invigorating and training the moral and intellectual faculties. The Pennsylvania system preserves the convict from contamination by evil communications with his fellow-prisoners, and prevents his associates from knowing the fact of his being in prison. These are advantages that go so far to compensate the evils of solitude, but do not remove them. In maintaining, as he does, that some men are moral patients who should be restrained, but not otherwise punished, he has often been met by the objection, that this doctrine destroys human responsibility. His answer has been, first, that in urging this view, he desires only to extend the class of idiots and the insane, who are by universal consent absolved from responsibility; and, secondly, that men in general, while they reject as dangerous and untrue the proposition in the abstract, adopt it practically, and are unwittingly guilty of the most flagrant inconsistency and pernicious injustice. Mr. C. has asked these objectors, if they would receive into their families, as domestic servants, or into their employment in stores, convicts who had served out their time in state prisons, supposing them qualified by knowledge for the duties of these stations; and most of them have answered that they would not. On being asked why they would decline, they have generally replied that they had not sufficient confidence in their reformation. There is obviously great inconsistency in such conduct. If they believe that every individual has power to reform himself, and that the prison is wisely framed to effect this reform, it is cruel to assume that the individual in question is not reformed, and to exclude him from social comfort and honour on this assumption. The truth is they *act* on the principle that some criminals are incorrigible, and that this may be one of the number, and therefore decline placing trust in any. Yet they blame him for teaching the same doctrine, and desiring to found on it a better practice.

Mr. Combe had the satisfaction of finding that these views of his are supported by the experience of the inspectors and warden of the "Eastern Penitentiary." They not only express a desire that the incorrigibles should be treated as patients, but strongly urge the necessity of an asylum for discharged convicts, previously to their returning into common society. They remark that the situation and sufferings of discharged convicts, have excited their attention and sympathy. The small sum of money—five dollars—allowed to a convict on his discharge is often expended whilst he is seeking for employment. But when that is gone, and no employment can be had, what hope is there that he will be able to struggle against poverty, and maintain his virtue? This class of men, as well as a large portion of the labouring poor, need advice and assistance to help them along the rugged pathway of life. The unwillingness manifested by most employers to take persons released from prison into their workshops, makes it difficult for convicts to obtain good situations at any period of the year, but during the Winter especially. Out-door work is scarce, and those discharged at this season often find themselves in so very destitute a situation, that we need not be surprised if they should sometimes be tempted to steal rather than starve. These officers believe much benefit would result from the courts either extending or diminishing in a slight degree the confinement, so as to make it terminate in either the Spring, Summer, or Autumn.

While this penitentiary system of treatment is pursued, it is manifestly necessary, in Mr. Combe's judgment, that there should be an asylum for convicts intermediate between the prison and society. Before a criminal can be fitted to re-enter the social circles of his country with a fair prospect of continuing in the paths of virtue, the discipline which he has undergone must have invigorated and enlightened his moral and intellectual powers to such an extent, that he, when liberated, shall be able to restrain his own propensities, amidst the usual temptations presented by the social condition. Now, there is only one way of strengthening faculties, and that is by exercising them ; and all the American prisons which he visited are lamentably deficient in arrangements for exercising the moral and intellectual faculties of their inmates. During the hours of labour, no advance can be made, beyond learning a trade. This is a valuable addition to a convict's means of reformation ; but it is not all-sufficient. After the hours of labour he is locked up in solitude, and it is very doubtful if he can read, for want of light ; but, assuming that he can—reading is a very imperfect means of strengthening the moral powers. They must be exercised, trained, and habituated to action. Mr. C.'s opinion is, that in prisons there should be a teacher of high moral and intellectual power for every eight or ten convicts ; that, after the close of labour, these instructors should commence a system of vigorous culture of the superior faculties of the prisoners, excite their moral and religious feelings, and instruct their understandings. In proportion as the prisoners give proofs of moral and intellectual advancement, they should be indulged with the liberty of social converse and action, for a certain time on each week-day, and on Sundays, in presence of the teachers ; and in these *conversaziones*, or evening parties, they should be trained to the *use* of their higher powers, and habituated to restrain their propensities. Every indication of overactive propensities should be visited by a restriction of liberty and enjoyment; while these advantages, and also respectful treatment, and moral consideration, should be increased in exact proportion to the advancement of the convicts in morality and understanding. By such means, if by any, the convicts would be prepared to enter society with their higher faculties so trained and invigorated, as to give them a chance of resisting temptation, and continuing in the paths of virtue. In no country, has the idea yet been carried into effect, that in order to produce moral fruits, it is necessary to put into action moral influences, great and powerful in proportion to the *barrenness* of the soil from which they are expected to spring. The convicts, whom he saw in this prison, presented the usual deficiencies in the organs of the moral sentiments in relation to those of the animal propensities which distinguish criminals in general.

We will now place on record some "Notes" of our traveller's, on the brain and skull in the savage and civilised families of mankind. Attended by several medical friends, he visited the Marine Hospital at Chelsea, near Boston, an institution for the temporary relief of sick and disabled seamen. All mariners, who have paid hospital-money, are admitted into it, except such as have contagious or incurable diseases, or who are insane. Here,

Mr. C. had an opportunity of witnessing two necrotomical inspections; and, at his request, Dr. Howe drew up an account of his observations on the skull and brain, in both subjects.

No. I. *Henry Nye,* a native of the Sandwich Islands: at eight years of age, he left his native island in a foreign ship, and passed his life as a sailor: he was a good-looking man, and a favourable specimen of his tribe; and his head presented the form which usually characterizes the Caucasian race: he died in his twenty-fourth year. When denuded of its membranes, his brain weighed exactly three pounds troy.

No. II. *Daniel Freeman,* a North American Indian: his parents were of the Gay Head tribe; but he lived principally among the whites, and had been on ship-board. He was a well-made man, with an organization superior to that of the generality of his tribe; he died in the hospital, aged twenty-nine years. His brain weighed two pounds, twelve and a half ounces.

According to Dr. Howe's notes, "both brains shewed a proportionately large development in the animal region: (Nye's the largest.) The organs of the moral sentiments were clearly the largest in Nye's brain: the anterior lobe was also longest, but they were of equal height. Nye's skull was higher and broader in the coronal region; it resembled more closely the Caucasian skull. The brain presented a corresponding development. Mr. Combe pointed out the greater development of the region of cautiousness in Nye's skull; and, on examining the corresponding convolutions of the brain, they appeared decidedly fuller than those in the brain of Freeman. The skull of Freeman was fuller in the region of veneration than Nye's; and the corresponding convolutions in the brain were also clearly larger. Nye's skull was more protuberant in the region of benevolence than Freeman's, and the corresponding convolutions of the brain were also fuller. The skulls of both were equally developed in the region of the organ of hope, and a corresponding fulness was observable in both, in the convolutions which constitute this organ. In general, the convolutions were rounder and plumper in the brain of Nye than in that of Freeman; but the latter was the firmer and harder of the two." From these and other instances, Mr. C. concludes that the natives of the Sandwich Islands possess a higher development of the moral and intellectual organs, in proportion to those of the animal propensities, than the North American Indians, and they have exhibited corresponding qualities of mind. They are more easily civilized and christianized.

On a former occasion, an "eminent philosophical divine" had drawn Mr. C.'s attention to the simplicity of the cranial sutures in *brutes,* and in the savage compared with the more civilized man: he had also remarked that, on the inspection of one quarter or less of a skull, he could decide whether it were that of a Charib, for instance, or a European; and that the complexity of the sutures in the latter attracts the notice of the most inexperienced eye. This led the party visiting the marine hospital to examine a variety of skulls, with a view to the determination of this point, and Dr. Howe recorded the following notes:—

"I. This was the skull of an Indian of the Gay Head tribe, aged fifty-six. The sutures were but faintly marked by a continuous line; the serrations had disappeared. The sagittal suture had disappeared entirely. II. A Penobscot

Indian's skull presented regular and distinct sutures. The serrations in the coronal suture were short, and not so minute as in the Caucasian crania. The serrations in the sagittal and lambdoidal sutures were distinct, and rather long, but not minute. III. A native of Celebes. The coronal suture presented no serrations; the bones seemed merely in juxta-position; and the dividing line was straight and distinct. The sagittal suture presented in the front part only a continuous straight line; in the back part a waving line, but no distinct serrations. The lambdoidal suture presented no regular serrations shooting distinctly across and into each other, but an irregular line. IV. A negro's skull presented a coronal suture with minute and distinct serrations; a sagittal and lambdoidal suture, with distinct and coarse serrations projecting across and far into each other. V. A Sandwich islander, aged twenty-four. The sutures were hardly discernible; the serrations not at all. VI. The skull of a North American Indian, aged twenty-nine, presented very faintly marked sutures with short serrations. The sutures not discernible on the inside of the skull."

These cases support the observation that the sutures are simple in savage skulls. Although we consider this fact in comparative osteology as established, yet would we recommend its farther verification, by frequent autopsy, in the hands of those naturalists who may find it practicable.

Our next note will present the somewhat singular feature of a *duad* bearing rather interestingly, and at the same time, on surgery and phrenology. We relate the case, as a little episode, and its consequences. It appears then, that Dr. Bush had informed Mr. Combe of Dr. M'Clellan's having removed two tumors from the head of a young man; one of them being internal to the skull, the other external, at the situation of the organs of firmness and conscientiousness. It was also stated, that a portion of the skull had been removed, to the extent of several square inches; that in this region, the brain was found to have disappeared; that, nevertheless, the patient had sat up in full possession of all his mental faculties, and conversed with the doctors during the operation; and that, particularly, he had manifested great firmness and self-possession. Having made this communication, Dr. B. requested Mr. C. to reconcile these facts with phrenology, as the case had attracted much attention, and was extensively spoken of, in Philadelphia, as one strikingly adverse to a fundamental principle of this science. Mr. Combe replied, with his characteristic straight-forwardness, that if such a case had occurred, it was the first sufficiently authenticated one which he had heard of; and that he could offer no opinion on it, without a personal inspection. Accordingly, with equal kindness and candour, Dr. M'Clellan took Mr. C. with him, when he went to dress the wound; observing, on the occasion, that the supposed difficulty in regard to phrenology had ceased to exist. He stated that, at the third dressing, to the astonishment of Dr. Bush and himself, "the cerebral convolutions had risen up; and that, in point of fact, they had never been destroyed, but only displaced by the pressure of the internal tumor." This was about the size and form of the half of a hen's egg cut longitudinally: the external tumor was nearly of the same size. They both arose from the effects of a blow inflicted with a stone, so slight at first as scarcely to be noticed; and their growth had extended over a period of three years. The interior tumor had been formed between the skull and the falx, the longitudinal canal having been carried down uninjured below its surface. Dr. M'Clellan remarked, "that the slow growth of these tumors explained the non-affection of the mental faculties; and that the brain had never been disorganized, but merely pressed downwards, and Nature had accommodated herself to the change." Dr. M'C. also stated, that this case shewed the importance of surgeons knowing accurately the situation of the organs; because, although he now saw that the organs of Firmness were not involved, he would at first have certified that they were destroyed, and that the patient manifested the faculty powerfully. He was now satisfied that the organs affected by the tumor were Self-esteem and Love of Approbation, and that he had not had adequate opportunities of judging whether the manifestations of these faculties were affected or not; besides, there was manifest evidence that the convolutions had not been disorganized.

Dr. M'Clellan's patient recovered; and, after his convalescence, he mentioned facts that shewed that his sentiments of Self-esteem and Love of Approbation had not remained unaffected during the progress of the disease. He was a player and ventriloquist, and performed in the western cities. He stated that, before receiving the blow, he was an entire stranger to diffidence. For the first three months after the accident, he felt no change in his mental condition, and was not aware that there was an affection of his head. At the end of that time, the external tumor began to attract his attention, and he felt also visitations of diffidence, which he had never before experienced. He was convinced that his powers of acting were unimpaired, yet he could not give effect to this conviction; for he felt as if he should fail. In the course of time, his self-confidence diminished so much, that he could no longer appear on the stage, yet his intellectual faculties were clear and active. So far, therefore, from this case being unfavourable to phrenology, it proved a striking confirmation of its truth, when all the circumstances had been fully investigated. Mr. Combe records the following honourable testimony to the magnanimity and ingenuousness of his transatlantic acquaintance, in these terms.

"Dr. M'Clellan is Professor of Surgery in the Jefferson College, Philadelphia, and he subsequently informed me, that before my arrival in that city, he had ridiculed phrenology in his lectures; that he had come to my lectures with the view of obtaining additional materials for refuting it; that he had at first conceived this case to be one strongly adverse to its pretensions, but now saw that it was the reverse; that the result of hearing my whole course, had been, to convince him of the futility of the objections on which he had previously relied, and to dispose him to devote a serious attention to the subject. Before Mr. C. left Philadelphia, the professor made the *amende honorable* to his class; told them that he had rashly condemned the science; that its principles were consistent with the best established facts in physiology, and that it was supported by a greater body of evidence than he had imagined. He wrote a letter to the same effect to Dr. Sewall of Washington, whom he had previously encouraged in his attacks against the science, and strongly counselled him to revise his opinions."

This Dr. Sewall published a book with the mis-title, "*The Errors of Phrenology refuted;*" but the refutations of the poor sciolist were soon triumphantly, though severely, refuted by Dr. Caldwell of Louisville, in his "*Phrenology Vindicated,*" where Dr. S.'s falsifications of the phrenological doctrine were shewn to be so flagrant, and his miserable sophistries to be so disreputable as to render him unworthy of credit as an evidence or of regard as a logician. In the "Notes," iii. 41, Mr. Combe exposes one egregious instance of this man's antiphrenological delinquencies, and then resigns him contemptuously to the judgment of posterity.

Mr. C. furnishes us with some insight into the "residences of the poor" in the Pennsylvanian capital. It is distressing to learn, he says, that even in this beautiful city, the houses of the poor too much resemble the residences of the same class in European towns. Dr. Parrish informed him that great numbers of young children die here every season in hot weather from *cholera infantum*, or, as it is commonly called, "the Summer complaint." The poor live in small houses, never intentionally ventilate their rooms, and seem not to know the use of cold water. The doctor would enter one of these dwellings on a summer morning when the thermometer stood at 90°, and find an infant shrivelled and bedewed with a clammy perspiration. It had been gasping all night for breath, and not drawn one mouthful of fresh air, and had, perhaps, never been washed from its birth. Death speedily relieves it. Many of the parents who thus treat their children are Irish. He hired an Irish nurse to suckle one of his own children. She gave her own son to an Irish family to board. When the hot weather came, he thought of her infant, and went to see it. It was in the condition before described. In three hours more it would have been dead. Without a day's delay, he sent the whole Irish family with the child to his farm, and saved it. "I should have felt very uneasy," said he, "if it had died, because my child was thriving under the care of the mother whom nature had given to it, but whom I had taken away for the benefit of my own."

Very many observations, both substantial and declamatory, have lately been published in this country regarding the treatment of insanity, with or without the accessory application of restraint. In one of Mr. Combe's "Notes," we meet with an affecting picture of the way in which the "insane poor" are still treated by our brethren in the West. By information derived from about half the counties of the State of Pennsylvania, a committee of the House of Representatives ascertained and reported that, in a population of nearly 800,000, there are upwards of 1100 insane persons, including congenital idiots and those rendered fatuous by disease; whence it is inferred, that in Pennsylvania at large, there is a total of 2300 insane and idiotic poor. Nearly two-thirds of this number are in the worst circumstances; and probably not less than a thousand of those unfortunates are kept in county poor-houses and prisons, or in families at auction-prices.* Not being subjected to medical or moral treatment, recovery under these circumstances is very rare, from five to eight in a hundred being an extremely favourable estimate. The following extracts from the committee's report shew the hardships to which these unhappy beings are exposed. "In one county of forty persons, more or less deranged, seven are confined in cells, which are nearly if not quite under ground. They may be seen from without through iron bars in the cellar windows.

* This means, that the poor are boarded out at so much a year to those who will take them at the cheapest rate.

Among them is a German girl, twenty years old, seemingly in perfect health of body, with beautiful teeth and hair, and without any symptoms of malignity, who has been in such a cell five months, and is considered as incurable. This interesting case, under treatment for a few months in a proper insane hospital, would probably result in a complete restoration to reason and liberty. Several other like cases are described, and all these, we are told, are shut up under bolts and bars, neglected and almost forgotten, with no friendly voice to break the silence of their solitude; and presented, one and all, the same revolting picture of suffering. In one county a man, thirty-five years old, had been confined for years in a miserable shed; when the bolt was drawn and the door opened, he was lying on the floor among straw, no bed was to be seen though it was cold weather, and we had to plunge through snow which had fallen the day previous, to get to his wretched abode. In another county, a woman of thirty-five was confined in like manner till she raved herself to death. While decided testimony is given to the good keeping and treatment of paupers generally, it is added that the poor lunatics are found with the feet chained together, or chained by the body to iron weights, logs of wood, or to the trunks of trees; or, what is more common, under ground, without ventilation, and breathing an air loaded with intolerable stench."

We are old enough to remember the time when an equal destitution both of humanity and science in the management of insane persons, was displayed very generally throughout the Old World, and there is reason still to fear that they do not experience so much tenderness of treatment, in some instances, as particular cases would justify. Dr. Dunglisson and other medical writers, have laboured zealously to free Pennsylvania from the disgrace of such scenes, and the forementioned committee have reported a bill for the erection of a proper asylum at the expense of the Commonwealth. We heartily wish it every success, in its erection, constitution and applications.

Ventilation, in all that regards its use and effects, belongs particularly to the province of medical investigation. Mr. Combe appears to possess a vivid and rational sense of its necessity, and he recurs no fewer than twenty-four times to the subject, with exhibitions of the evils resulting from its neglect, and of the advantages derivable from its proper and efficient regulation. With an outline of his "Notes" and illustrations of this process, so indispensable to the health of mankind, in all climates and at all temperatures, we shall conclude the present article. Mr. C. was in the habit we perceive, of extending his prelections to an unusual length; but he adopted this practice under precautions which insured to his hearers the benefits of fresh air and change of posture. We discern, in his practice, a system which might be usefully adopted by professional lecturers and their pupils. He opens one of his "Notes," relating to ventilation, with the remark: a sermon of an hour's duration appears very long, and a lecture of two hours wears a still more formidable and forbidding aspect. Aware of this, he delivered at the end of the first hour a brief address, by way of episode, to the audience, mentioning that phrenology taught us that the mind thinks by means of the brain, just as we walk by means of the legs; that the brain is liable to become fatigued by too long attention, as the locomotive muscles are by too much walking; and he therefore proposed to them to take a brief rest. He requested them to stand up in order to vary their position, also to converse freely with each other for the sake of relaxation, the more merrily the better, for cheerfulness circulates the blood; and he called their attention also to the absence of all means of ventilating the hall, remarking that, as we had already breathed the air which it contained for a full hour, it must have lost much of its vital properties, and needed to be renewed. He requested the gentlemen to put on their hats, and the ladies their shawls, to avoid catching cold, and then had the windows widely opened. This proceeding caused some astonishment and alarm at first; for the Americans generally have a dread of cold air, amounting almost to an aërophobia. He assured them that they would suffer no inconvenience, and they submitted to the experiment. The interval allowed was only five minutes, at the end of which he resumed the lecture; but so refreshing were the effects of the brief rest, of the change of position, and, above all, the admission of pure air, that during the second hour the attention was as completely sustained as during the first. The same practice was continued every evening through the whole course, and with the same success. Many individuals expressed their gratification at having discovered such simple means of relieving the tedium of a long discourse; and as his audience continued to increase, after the length of the lectures was generally known, it became evident that the two hours' application, when thus arranged, was not felt as an unbearable affliction.

Many of the schools, at Boston, are deficient in ventilation; and, says Mr. C., the consequence of this are headache, loss of appetite, and irritability, in such of the teachers as do not enjoy exceedingly robust constitutions; and drowsiness in the children, in the latter portion of each meeting, when the air is particularly foul. In the morning, when the children come fresh to school, they look healthy, cheerful, and well dressed; but words form the staple of the instruction, to the too great neglect of objects. Improvement in the things taught, as well as in the modes of teaching, advances slowly, not through want of good attention in the members of the school committees, but from attachment to old customs, and lack of knowledge of better modes than those now existing. The necessity of ventilating common-school houses however has been efficiently recognised at Boston in a new erection of the kind. Mr. C. visited it, and thus describes the arrangements. The ceilings of the rooms are high. In Winter a large supply of air, heated by a brick furnace to a moderate temperature, is introduced, and it is let off by five or six separate flues in different parts of the room, which can be opened and shut at pleasure. In each of the rooms the temperature, regulated by a thermometer, was 67° F., the external air being 35°. The garret which used to be lost, has, at Dr. Howe's suggestion, been floored and plastered, and furnished with swinging ropes; and in bad weather the children play in it during the intervals of teaching. All the seats have backs. The teachers told me, that since they have occupied this school-house, the vivacity and capacity of the scholars have obviously been raised, and their own health and energy increased. Appended to this, he transcribes from a tract intituled "Reasons for establishing a Society for improving the Dwellings of the Labouring Classes in Edinburgh, 1840," the following statement:—"A

metal tube, opening from the upper part of the wall of the room, and joining a general tube which terminates in the furnace of some neighbouring factory, is all that is required to ensure a constant supply of fresh air to the inmates of that chamber, though, as often happens, they should be upwards of a dozen in number. A few years ago, a large building in Glasgow, each room of which contained a family, and the tenants of which were in all five hundred, was ventilated in this way, and the result was most satisfactory. Previously to the ventilation, diseases, and particularly typhus fever, had been very fatal to the inmates; five persons had been ill of the latter disease in one room, and in two months, at the end of 1831, fifty-seven had been attacked by it. After the apparatus was applied, four and a half years elapsed, during which there were only three cases of fever, and two of these in a room where the tube had been destroyed. Mr. C. earnestly recommends these facts to the attention of the Americans of all classes; for they are little sensible of the extent to which they injure themselves by living in bad air.

In one of his phrenological lectures, when treating of "Physical Education," Mr. C. embraced the opportunity to inculcate the indispensableness of a healthful atmosphere to the preservation and improvement of our native energies, both physical and mental. Having observed the unwholesome condition of the class-rooms, court-rooms, and other places of public resort in Boston, for want of ventilation, he called the attention of the audience strongly to the dependence of the mental faculties on the condition of the brain for their power of action; to the dependence of the brain, for its vital properties on the condition of the blood; and to the dependence of the blood on the condition of the digestive and respiratory organs; thus pointing out the direct connection between sound digestion, pure air, and mental vigour. He found that even a brief exposition of the structure and functions of the digestive and respiratory organs, and of their connection with the brain, illustrated by large drawings, brought home to the understandings of his audience the importance of digestion and ventilation to mental energy, and gave general satisfaction. These ideas were by no means new to them; but, although they had often heard them stated by other lecturers, and had read them in books, it had occurred to few to carry them into practice. He, therefore, insisted largely on the evils which they inflict on themselves and their children by this neglect. Pulmonary consumption produces a large proportion of all the deaths that occur in New England, and he pointed out to them the train of causes in full operation, which lead to this disease. By breathing hot and vitiated air in ill ventilated apartments, the blood is not properly aërated, the lungs are enfeebled, and the tone of the whole system, mental and bodily, is lowered; nevertheless in this condition they make the most rapid transitions from a temperature of 70° or 75° of Fahrenheit's thermometer, which is common in their houses, churches, and lecture-rooms, to one of 5 or 10 degrees below zero, in the open air; a change sufficient to injure the respiratory organs in the most robust state of health, and much more so when weakened by this previous injudicious treatment.

On his visit to the "Farm Schools," Mr. C. thought the children presented a melancholy aspect. The weather was cold, and as the cold had come on suddenly many of them had not yet received their winter supply of stockings and shoes. They were crowding round the stoves with an expression of suffering and discomfort, which was distressing to behold. The buildings in which they live, are frame or wooden houses, divided into moderate-sized rooms, low in the ceilings, and without any means of ventilation except the doors and the windows. They sleep crowded together in these apartments ; the beds stand so close to the windows and the air is so cold, that they are not open during the night, and the air is excessively vitiated before the morning. The consequences are visible in the appearance of the children ; many of them are suffering under ophthalmia, and they present generally that sunken, inanimate, and unhappy aspect which betokens blood in a bad condition from imperfect nutrition and impure air. There is, he believes, no stinting of food ; but the digestive functions suffer from the confinement in an unwholesome atmosphere, and hence the nutrition is imperfect. Speaking of the Alms-House at Philadelphia, which is commonly denominated the "Pauper Palace," from its being altogether so magnificent in reference to its objects, our author relates, that the whole establishment is kept clean to the eye, but the nose and lungs detect imperfect ventilation, particularly in the departments for the children ; who are afflicted with ophthalmia, languid looks, and other indications of a low condition of the corporeal system. It is extremely difficult to induce paupers voluntarily to admit fresh air into their apartments, except in very warm weather, and in building an alms-house, adequate means for involuntary ventilation as well as warmth should be provided. He was glad to observe that pictures, objects, and apparatus, are supplied for teaching the children ; an advantage not enjoyed in many of the city schools. Mr. C. attended a public meeting in the Hall of the Supreme Court, and he complains of its being destitute of ventilation. He suffered severely for several hours after leaving it from the effects of bad air. On mentioning this next day, he was told that several lawyers have fallen down dead on the spot while engaged in the most animated pleadings in this hall, and that, although apoplexy was assigned as the cause, some medical men, who knew the state of the atmosphere, had expressed an opinion that these catastrophés were probably hastened if not caused, by asphyxia. The late arrangements for ventilating the British Houses of Parliament are well known here ; but no person has yet proposed to adopt them, or any other means, for the preservation of life and health in the public chambers and apartments of the capital.

Throughout this long article, we have not been obtrusive, we trust, with a too frequent introduction of phrenological facts and inductions: with this instance of self-denial for our plea, therefore, we would claim the reader's indulgence while we follow Mr. Combe in his excellent remarks on "phrenology and education :" the last being a theme on which the anxieties of our profession are, in these days, intensely concentrated. The observation was occasionally made to Mr. C. by persons who had heard his lectures on education, without having attended those on phrenology, that the views presented were so sound and luminous that he would have done much more good if he had omitted phrenology, and delivered them simply as founded on common sense. This, said they, would have saved the lectures from the prejudices which exist in so many minds against phrenology, and which render them suspicious of every doctrine and practice springing out of it. His answers were, *first*, that a knowledge of the influence of the organs on the power of manifesting the mental faculties, is a fundamental requisite to the right understanding of the subject of education. *Secondly*, That to have withheld this important knowledge, because it was unpopular, would have been improper and uncandid. By following such a course he should also have been extending the impression already produced by too many disingenuous phrenologists, that the science is worthless, and that the soundest views of education may be obtained without its aid, which he knows not to be the case. *Thirdly*, That such conduct would have been unjust and injurious towards the founders and defenders of phrenology. It would have been appropriating to himself the fruits, and leaving to them not only the toil but the obloquy of having raised them. *Fourthly*, That lectures on education, founded on phrenology, make a deeper and more permanent impression on the understanding than if based on mere common sense, and can be more certainly and successfully carried into practice. Every man's common sense differs from that of his neighbour. In New England, he had visited a school, the head master of which told him, that he devoted one half of his whole hours of teaching to arithmetic and mathematics, because he had discovered that pupils who excelled in those branches soon became proficients in every other, such as grammar, geography, and repetitions. No phrenologist could have held such views, because he must have known that arithmetic and mathematics depend on different faculties from those which take cognizance of language, grammar, and general reasoning. Mr. C. observed that the organs on which arithmetic and mathematics depend predominated over the whole intellectual organs in this person's own head; in consequence of which he could teach these branches with most ease and success, and his common sense led him to conclude that all his pupils were similarly constituted to himself. When teachers rely solely on common sense and their own experience, they act merely on the suggestions of their strongest propensities, sentiments, and intellectual faculties, whatever these may be, without reference to the differences which exist between their minds and those of their pupils. Phrenology presents a scientific guide to all.

With this miscellany of selections, we must now take leave of Mr. Combe's *Moral Philosophy* and his *Notes on the American People*, their manners, character, and institutions. His pilgrimage through the transatlantic states was chiefly a "Phrenological Visit," during which he registered a multitude of curious and most interesting facts relating to the physiology of the brain in connexion with the manifestations of the mind; and, in the reports of his observations, the phrenologists will be gratified to find that the diffusion of their science is proceeding rapidly among the intelligent and enlightened classes of American citizens. Both in his *Philosophy* and his *Notes*, the general reader will meet with many speculations and principles strongly calculated to suggest the gravest reflections to a mind exercised in determining the preferable structure of society, and its bearings on the well-being of individuals and families and nations. In his greatly diversified topics relating to ecclesiastical polity, the theologian and legislator may discover the elements of a fair comparison between the advantages derivable from an established national church and those of chapels for worship supported by voluntary contributions—between the patriotism of a limited monarchy and that of a democracy. Here we have accumulated a medley of sketches valuable, as we deem them, and appropriate as a study for the medical and ethical inquirer. For ourselves, we are not able to accept of Mr. C.'s guidance in the momentous concerns of politics and religion; but we greatly applaud the purity and fervour of his philanthropy, and we venerate with a grateful devotion his philosophy of the human mind, with its beautiful and unbounded and benignant applications.

